# Prediction of Early Recurrence of Liver Cancer by a Novel Discrete Bayes Decision Rule for Personalized Medicine

**DOI:** 10.1155/2016/8567479

**Published:** 2016-10-09

**Authors:** Hiroyuki Ogihara, Norio Iizuka, Yoshihiko Hamamoto

**Affiliations:** ^1^Department of Biomolecular Engineering, Graduate School of Medicine, Yamaguchi University, Tokiwadai 2-16-1, Ube, Yamaguchi 755-8611, Japan; ^2^Department of Kampo Medicine, Graduate School of Biomedical & Health Sciences, Hiroshima University, 1-2-3 Kasumi, Minami-ku, Hiroshima 734-8551, Japan; ^3^Division of Electrical, Electronic and Information Engineering, Graduate School of Sciences and Technology for Innovation, Yamaguchi University, Tokiwadai 2-16-1, Ube, Yamaguchi 755-8611, Japan

## Abstract

We discuss a novel diagnostic method for predicting the early recurrence of liver cancer with high accuracy for personalized medicine. The difficulty with cancer treatment is that even if the types of cancer are the same, the cancers vary depending on the patient. Thus, remarkable attention has been paid to personalized medicine. Unfortunately, although the Tokyo Score, the Modified JIS, and the TNM classification have been proposed as liver scoring systems, none of these scoring systems have met the needs of clinical practice. In this paper, we convert continuous and discrete data to categorical data and keep the natively categorical data as is. Then, we propose a discrete Bayes decision rule that can deal with the categorical data. This may lead to its use with various types of laboratory data. Experimental results show that the proposed method produced a sensitivity of 0.86 and a specificity of 0.49 for the test samples. This suggests that our method may be superior to the well-known Tokyo Score, the Modified JIS, and the TNM classification in terms of sensitivity. Additional comparative study shows that if the numbers of test samples in two classes are the same, this method works well in terms of the *F*1 measure compared to the existing scoring methods.

## 1. Introduction

Liver cancer is one of the refractory cancers, and overcoming it is of national concern. Despite complete surgical resection, the problem with refractoriness lies in the high percentage of recurrences of liver cancer [1]. If recurrence can be accurately predicted for each patient, effective treatment can be administered, and recurrence-free patients may not need to be given unnecessary anticancer agents or undergo computed tomography (CT) scans. As a result, health care costs can also be controlled. Therefore, personalized medicine that provides the best cancer treatment to each patient is needed.

The difficulty with cancer treatment is that even if the types of cancer are the same, the cancers vary depending on the patient. Even a wide variety of tests such as blood tests or CT scans reveal only certain aspects of cancer, and there is no test that can perfectly detect cancer. Nevertheless, by using various combinations of laboratory data (also called markers), the Tokyo Score [2], the Modified JIS [3], and the TNM classification [4, 5] have been proposed as representative liver scoring systems. However, none of these scoring systems have met the needs of clinical practice. The reason is that as noted above, each marker has not played a decisive role in the prediction of recurrence, and further, the combination of markers used in these scoring systems has been experimentally obtained from the results of physician trial and error, indicating no assurance of optimal prediction.

The diagnosis of leukemia was made possible using levels of gene expression, and this triggered the diagnosis of cancer by machine learning [6]. As a result, full-scale deployment of microarray techniques has begun in the field of cancer diagnosis. Furthermore, thanks to the latest progress in machine learning such as artificial neural networks [7] and support vector machines (SVM) [8], various cancer diagnostic methods have been developed [9–14]. Other than these techniques involving levels of gene expression, reports have been published on the diagnosis of liver cancer by blood tests that characteristically use methylation volume [15] and on the diagnosis of lung cancer through artificial neural networks that characteristically use clinical data and which are based on SVM [16]. However, all of these methods use quantitative data that consequentially limit the available data.

Generally, with laboratory data, quantitative data (metric data or continuous data), which are represented by numerical numbers, intermingle with qualitative data (nonmetric data or categorical data). Unfortunately, as with the machine learning described above, the conventional Bayes classifier, which is particularly popular in statistical pattern recognition [17], cannot also be applied to qualitative data. To overcome this practical limitation, we propose a discrete Bayes decision rule that deals with qualitative data. For the quantitative data, they are first converted to qualitative data by thresholding, and as a result, all laboratory data become discretized data (qualitative data). Then, a patient is represented as a pattern vector that consists of the discretized laboratory data. Next, the problem of predicting recurrence is defined as a two-class problem that distinguishes between a pattern of recurrence class and a pattern of nonrecurrence class in a patient. For this two-class problem, a classifier is designed according to our own discrete Bayes decision rule that can handle discrete data, and the resulting classifier distinguishes between the presence and absence of recurrence with a high degree of accuracy. Moreover, to handle the high dimensionality of pattern vectors, the optimal combination of markers is selected by feature selection, which is based on the resampling technique using virtual samples.

## 2. Decision Rule and Feature Selection

### 2.1. Classifier Design by Discrete Bayes Decision Rule

As previously mentioned, the discrete Bayes classifier is characterized by the fact that it can handle discrete data. Given *M* markers as candidates, the range of each marker is divided into divisions so that they become mutually exclusive events. Suppose that *x*_1_, *x*_2_,…, *x*_*d*_ are selected as *d* targeted markers from among *M* markers and then the discretized laboratory data of a patient in marker *x*_*j*_ belongs to the division *x*_*j*(*r*_*j*_)_, *j* = 1,2, …, *d*. Here, *x*_*j*(*r*_*j*_)_ denotes the *r*_*j*_th division of marker *x*_*j*_. The subscript *j* is a discrimination number representing a marker. Then, the patient is described as the pattern vector **x** = [*x*_1(*r*_1_)_, *x*_2(*r*_2_)_,…, *x*_*d*(*r*_*d*_)_]. The class-conditional probability *P*(*x*_*j*(*r*_*j*_)_∣*ω*_*i*_) of the division *x*_*j*(*r*_*j*_)_ for class *ω*_*i*_ is defined as

(1) $$\begin{array}{c} P\left(\;x_{j\left(r_{j}\right)}\;|\;\omega_{i}\;\right)=\frac{n_{j\left(r_{j}\right)}^{i}}{\sum_{k=1}^{d}n_{k\left(r_{k}\right)}^{i}},\;j=1,2,\ldots ,d, \end{array}$$

where *n*_*j*(*r*_*j*_)_^*i*^ represents the number of patients who belong to the division *x*_*j*(*r*_*j*_)_ among *n* ^*i*^ patients from class *ω*_*i*_.

Assuming that, in general, the events in which the discretized laboratory data belong to any of the divisions are mutually independent, the class-conditional probability *P*(**x**∣*ω*_*i*_) is described as follows:

(2) Px ∣ ωi=Px1r1,x2r2,…,xdrd ∣ ωi=Px1r1 ∣ ωiPx2r2 ∣ ωi⋯Pxdrd ∣ ωi=∏k=1dPxkrk ∣ ωi.

The posterior probability *P*(*ω*_*i*_∣**x**  )  in the two-class problem is provided by Bayes' theorem as follows:

(3) $$\begin{array}{c} P\left(\;\omega_{i}\;|\;x\;\right)=\frac{P\left(\omega_{i}\right)P\left(x\;|\;\omega_{i}\right)}{P\left(\omega_{1}\right)P\left(x\;|\;\omega_{1}\right)+P\left(\omega_{2}\right)P\left(x\;|\;\omega_{2}\right)}, \end{array}$$

where *P*(*ω*_*i*_) is the prior probability for class *ω*_*i*_. To equally deal with the recurrence class and nonrecurrence class, we assume that the prior probability *P*(*ω*_*i*_) is an equal probability of 0.5. Then, the posterior probability is simplified as follows:

(4) $$\begin{array}{c} P\left(\;\omega_{i}\;|\;x\;\right)=\frac{P\left(x\;|\;\omega_{i}\right)}{P\left(x\;|\;\omega_{1}\right)+P\left(x\;|\;\omega_{2}\right)}. \end{array}$$

By substituting (2) into this formula, the posterior probability can be calculated as follows:

(5) $$\begin{array}{c} P\left(\;\omega_{i}\;|\;x\;\right)=\frac{\prod_{k=1}^{d}P\left(x_{k\left(r_{k}\right)}\;|\;\omega_{i}\right)}{\prod_{k=1}^{d}P\left(x_{k\left(r_{k}\right)}\;|\;\omega_{1}\right)+\prod_{s=1}^{d}P\left(x_{s\left(r_{s}\right)}\;|\;\omega_{2}\right)}. \end{array}$$

By the discrete Bayes decision rule, a pattern **x** is classified into a class *ω*_*i*_ where the posterior probability *P*(*ω*_*i*_∣**x**) is maximum.

Cases of the relationship between the divisions and the number of patients are shown in Table 1. Table 1 shows that the range of marker *x*_2_ is divided into three divisions of *x*_2(1)_, *x*_2(2)_, and *x*_2(3)_, and among these divisions the division *x*_2(2)_ includes *n*_2(2)_^*i*^ patients from class *ω*_*i*_. Here, the total sum of the number of the patients in each division is the following: *n*_2(1)_^*i*^ + *n*_2(2)_^*i*^ + *n*_2(3)_^*i*^ = *n* ^*i*^. As previously mentioned, *n* ^*i*^ is the number of all patients from class *ω*_*i*_.

**Figure 1:**
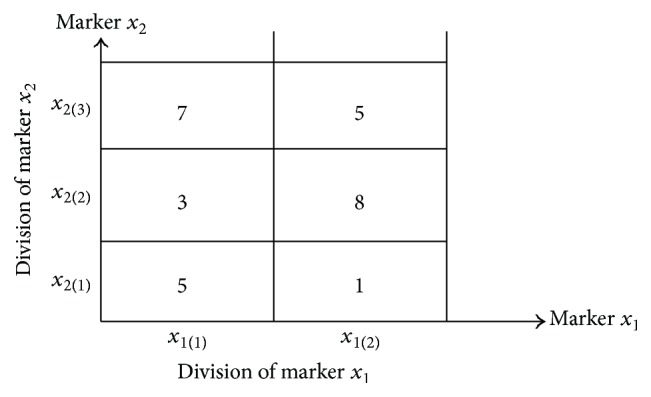
Arrangement of training samples of the recurrence class for markers *x*_1_ and *x*_2_.

We explain the discrete Bayes decision rule by using concrete cases. Assume that in the case of *d* = 2 markers *x*_1_ and *x*_2_ are used and the discretized laboratory data of a patient belong to *x*_1(1)_ in marker *x*_1_ and *x*_2(3)_ in marker *x*_2_.  Figure 1 illustrates this with the use of data from Table 2 and shows that the number of recurrence patients belonging to *x*_1(1)_ and *x*_2(3)_ is 7. Then, the equations are the following:

(6) Px11 ∣ ω1=n111n111+n231,Px23 ∣ ω1=n231n111+n231,

and the class-conditional probability *P*(*x*_1(1)_, *x*_2(3)_∣*ω*_1_) is as follows:

(7) $$\begin{array}{c} P\left(\;x_{1\left(1\right)},x_{2\left(3\right)}\;|\;\omega_{1}\;\right)=P\left(\;x_{1\left(1\right)}\;|\;\omega_{1}\;\right)P\left(\;x_{2\left(3\right)}\;|\;\omega_{1}\;\right). \end{array}$$

Similarly, *P*(*x*_1(1)_, *x*_2(2)_∣*ω*_2_) is obtained and from (5) and the posterior probabilities of classes *ω*_1_ and *ω*_2_ are each obtained, and then the patient is classified into the class in which the posterior probability is maximum.

Now, let us compare the discrete Bayes classifier to the conventional Bayes classifier, both of which classify patterns based on the posterior probability. In the conventional Bayes classifier, a pattern is represented as a multidimensional vector that consists of quantitative data, and the statistical information of the pattern distribution is in the mean vector and covariance matrix. In general, the number of patients is small and the number of dimensions is large. In small sample size situations, the discrete Bayes decision rule is also influenced by the number of samples, as with the conventional Bayes decision rule, and the discrimination ability deteriorates. In the conventional Bayes decision rule, an inverse of a covariance matrix may not exist. At this time, although the conventional Bayes classifier cannot be designed, the discrete Bayes classifier can be designed, irrespective of an inverse matrix. The first advantage of the discrete Bayes decision rule is that it does not need the inverse of a covariance matrix. Second, from the viewpoint of computational cost, in the conventional Bayes classifier, which deals with quantitative data alone, the computational cost increases sharply with an increasing number of dimensions (number of markers). Meanwhile, in any of the discrete Bayes classifiers, which deal with discrete data alone, computation is only scalar computation. Even if a dimension is high, the discrete Bayes classifier can be easily calculated, indicating that the classifier is practical. To simplify the discussion, we have so far dealt with a two-class problem, but this decision rule can easily be extended to multiclass problems.

### 2.2. Selection of Optimal Markers

**Figure 2:**
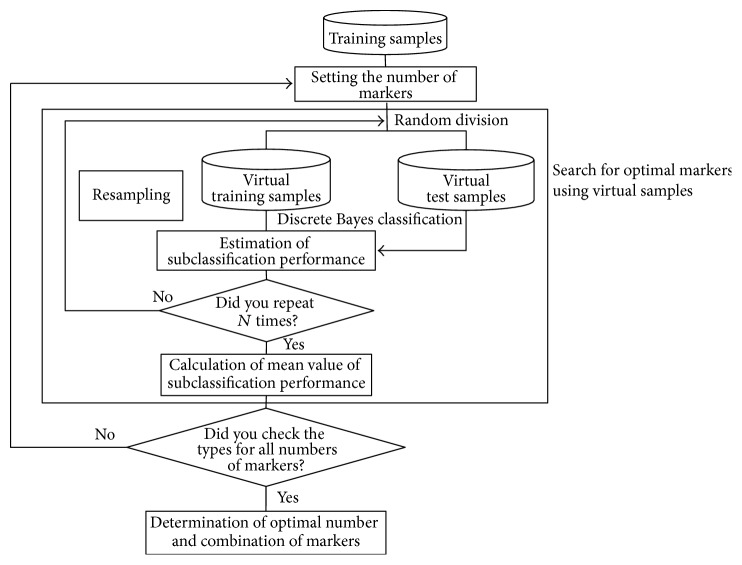
Selection of optimal markers.

 Knowing which markers are used for the discrete Bayes classifier is essential in discrimination. This is a problem of feature selection in the statistical pattern recognition fields [17]. Here, we explain a method to solve a feature-selection problem in which a combination of* d* markers useful for discrimination is selected from among *M* candidate markers. The small number of training samples in marker selection often causes the overfitting problem [18] that while a classifier with use of selected markers may allow perfect classification of the training samples, it is unlikely to perform well on new patterns. To avoid the overfitting problem, by using virtual samples, which are produced by the resampling technique from available training samples, an optimal combination of markers is obtained. This idea comes from a Bootstrap technique [19]. The flow is shown in Figure 2.

First, training samples are randomly divided into virtual training samples and virtual test samples. Then, the number of markers at the start of searching is determined, and one combination of markers of interest is used. Second, based on the discrete Bayes decision rule with the markers of interest, the class-conditional probability *P*(**x**∣*ω*_*i*_) is calculated from the discretized laboratory data of the virtual training samples, and a virtual test sample is classified based on the posterior probability *P*(*ω*_*i*_∣**x**) calculated by using the class-conditional probability. In discrimination, we investigate which divisions the discretized laboratory data of a virtual test sample belong to according to the markers of interest. Next, using the class-conditional probability corresponding to the divisions to which the data belong, the posterior probability is obtained from (5), and the virtual test sample is classified into a class in which the posterior probability is maximum. The procedures described above are repeated independently *N* times. The means of the sensitivity and specificity against the virtual test samples are estimated from *N* trials. Then, using the same number of markers, the whole of another combination of markers is evaluated. Because we consider that the prediction of recurrence is important, high sensitivity is required. Now, in the number of markers involved, under the constrained conditions of a specificity of 0.5 against the virtual test samples, a combination of markers that maximizes the mean sensitivity is selected as a candidate of the optimized solution for the number of markers involved. Next, the number of markers is increased by one, and this similar procedure is repeated until the number of markers *M* − 1 is reached. Thus, from among the candidates obtained, an optimal combination of markers is selected and is used for the design of the classifier.

## 3. Experiment

### 3.1. Data

Data were obtained from patients whose liver cancers were entirely excised during surgery at Yamaguchi University School of Medicine. Of these patients, 57 experienced a recurrence of liver cancer within one year and 177 experienced no recurrence. Liver cancer is classified as type C liver cancer, type B liver cancer, and others depending on the infecting virus type. The virus types of liver cancer used are shown in Table 3 according to the training samples and the test samples. Additionally, similar to the Tokyo Score, Modified JIS, and TNM classification, the proposed method is also not reliant on virus type.

Among candidate markers such as ALB, tumor number × tumor size [20], ICG, vp, vv, platelets, PT, bilirubin, degree of differentiation, and liver damage, an optimal combination of markers was obtained. This optimal combination was used for the discrete Bayes classifier.

We explain the details of Table 2, in which the cutoff value for each marker was determined by a physician in advance, as follows. As an example, for ALB, patients are divided into patients with recurrence and patients without recurrence based on whether the value of the marker exceeds 3.5. Among 29 of the patients with recurrence, 15 have an ALB of greater than 3.5, and 14 have an ALB of not more than 3.5. For any marker, the total number of divided recurrence patients is 29 within the marker.

### 3.2. Experimental Method

**Figure 3:**
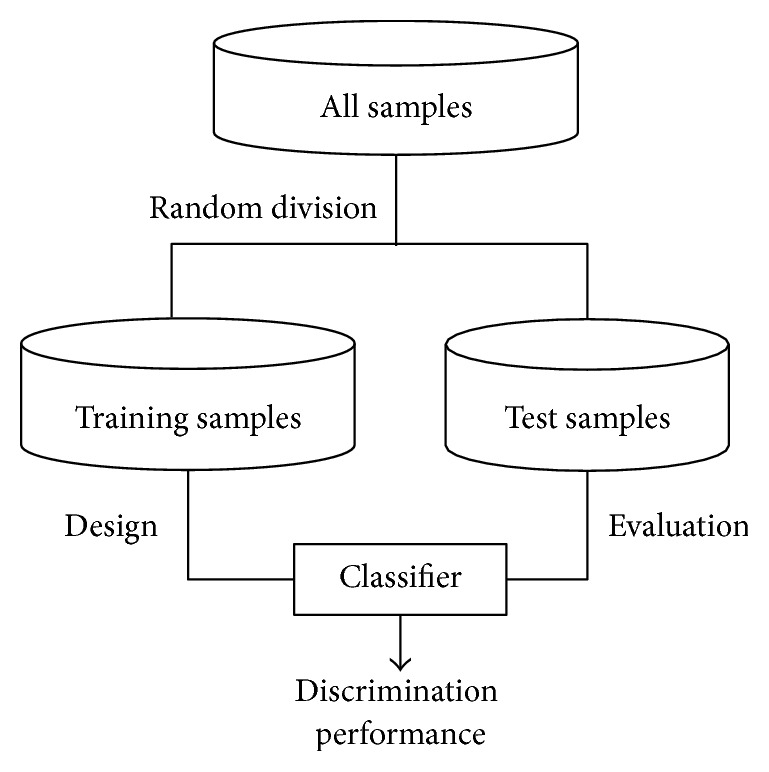
Flow of classifier design and evaluation.

The holdout method [21] splits the available data into two mutually exclusive sets, referred to as the training and test sets. The classifier is designed using the training set, and performance is evaluated on the independent test set. The holdout method preserves the independence of the training samples and test samples, which are used for estimation of discrimination performance. The recurrence samples (*n* = 57) and nonrecurrence samples (*n* = 177) are each randomly divided into halves, and one-half is assigned to training samples and the other half to test samples. Consequently, the number of recurrence training samples is 29 and that of nonrecurrence training samples is 89, whereas the number of recurrence test samples is 28 and that of nonrecurrence test samples is 88. The flow of classifier design and evaluation is shown in Figure 3. An optimal combination of markers was determined initially.

**Figure 4:**
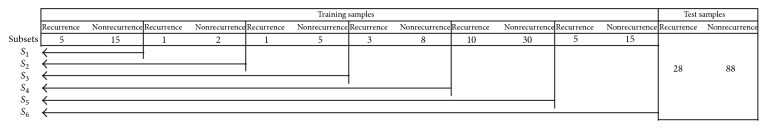
Relationships between the training sample subsets.

Next, the optimal combination of markers was fixed, and the number of training samples is discussed. Generally, an increase in the number of training samples results in an improvement of the discrimination ability of the classifier. Therefore, a designer is interested in how many training samples are needed for classifier design. Then, as a subset of the increased number of training samples, we assume that a series of 6 training subsets, as shown in Figure 4, holds the following relation:

(8) $$\begin{array}{c} S_{1}\subset S_{2}\subset S_{3}\subset S_{4}\subset S_{5}\subset S_{6}. \end{array}$$

Here, a subset takes a structure of nested subsets. The first subset *S*_1_ consists of 5 recurrence training samples and 15 nonrecurrence training samples. The next subset *S*_2_ contains 23 training samples by adding one true recurrence training sample and two nonrecurrence training samples. In this manner, a discrete Bayes classifier is designed for each subset with increased true training samples, and the discrimination abilities of 6 discrete Bayes classifiers for the same test samples are obtained. This trial was independently repeated 30 times, and the influence of an increase in training samples on discrimination ability was investigated.

Then, a comparison of the performance of the classifiers with the existing scoring formulae was conducted. For comparison, we adopted accuracy, sensitivity, specificity, the Youden index [22] (= sensitivity + specificity − 1.0), *F*1 measure, and diagnostic odds ratio [23, 24] for the test samples as evaluation values of the discrimination performance of a classifier. The higher the values are, the higher the discrimination performance of a classifier is. Here, sensitivity means the rate of correctly classifying patients as patients with recurrence among all patients with recurrence, and specificity means the rate of correctly classifying patients as patients without recurrence among all patients without recurrence. To predict early recurrence, our primary interest is in the sensitivity, under the constrained condition of a specificity of 0.5. ROC analysis [18] was also performed.

Here, we explain the score formulae as targets for comparison. For the Tokyo Score [2], the Modified JIS [3], and the TNM classification [4, 5], a score value is assigned to each marker used according to cutoff value determined by a physician. Then, a total score is assigned as a summation of all individual score values. Patients are diagnosed by the cutoff value against this summary score. For example, in the Tokyo Score, 4 markers, albumin, bilirubin, tumor size, and the number of tumors, are used. If a patient has an albumin value of 3.0 g/dL, bilirubin value of 1.5 g/dL, tumor size of 1.0 cm, and the number of tumors of 4, the score values of each marker are 1 point, 1 point, 0 points, and 2 points, respectively, resulting in a total score of 4 points. If the cutoff value is 2, then the patient is diagnosed as having possible recurrence.

Finally, as described previously, the discrete Bayes classifier uses scalar function alone for discrimination, and, thus, it has a computational advantage. To clarify this advantage, we prepared 1,160,000 artificial test samples that were obtained from 116 actual test samples copied 10000 times. Then, using combinations of the markers shown in Table 4, the discrimination time of the artificial test samples was measured by changing the number of markers one by one from 3 to 6. The discrimination time was defined as the time from the start to the end of the discrimination process, and the time was measured using the clock function.

### 3.3. Results

#### 3.3.1. Optimal Combinations of Markers

Table 4 shows the candidate combinations of the markers that were obtained from 100 times of resampling per number of markers and their discrimination performances. A dash symbol in the table indicates that no combinations meeting the constrained conditions of sensitivity and specificity existed. Based on sensitivity under the constrained condition of a specificity of 0.5, a combination of the four markers of tumor number × tumor size, vp, ICG, and liver damage was considered to be optimal, among the four candidates.

#### 3.3.2. Influence of the Number of Training Samples on Discrimination Performance

**Figure 5:**
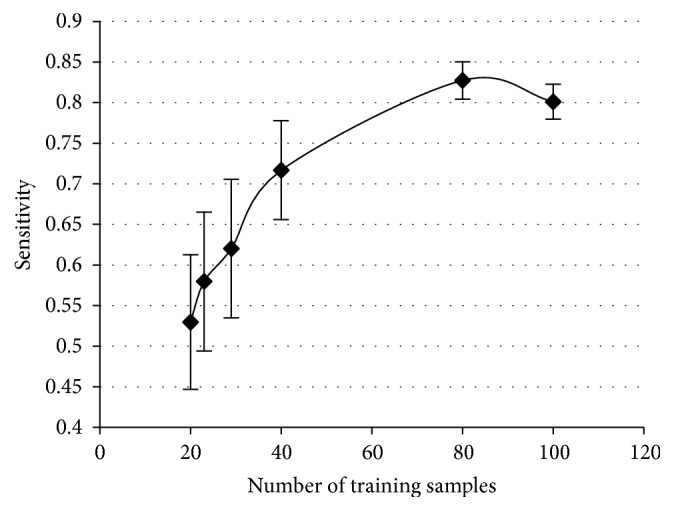
Relationship between the number of training samples and sensitivity.

The relationship between the number of training samples and sensitivity is shown in Figure 5. The horizontal lines indicate the 95% confidence intervals of the sensitivity values.

#### 3.3.3. Performance Comparison between the Classifier and Existing Liver Scoring Systems

**Figure 6:**
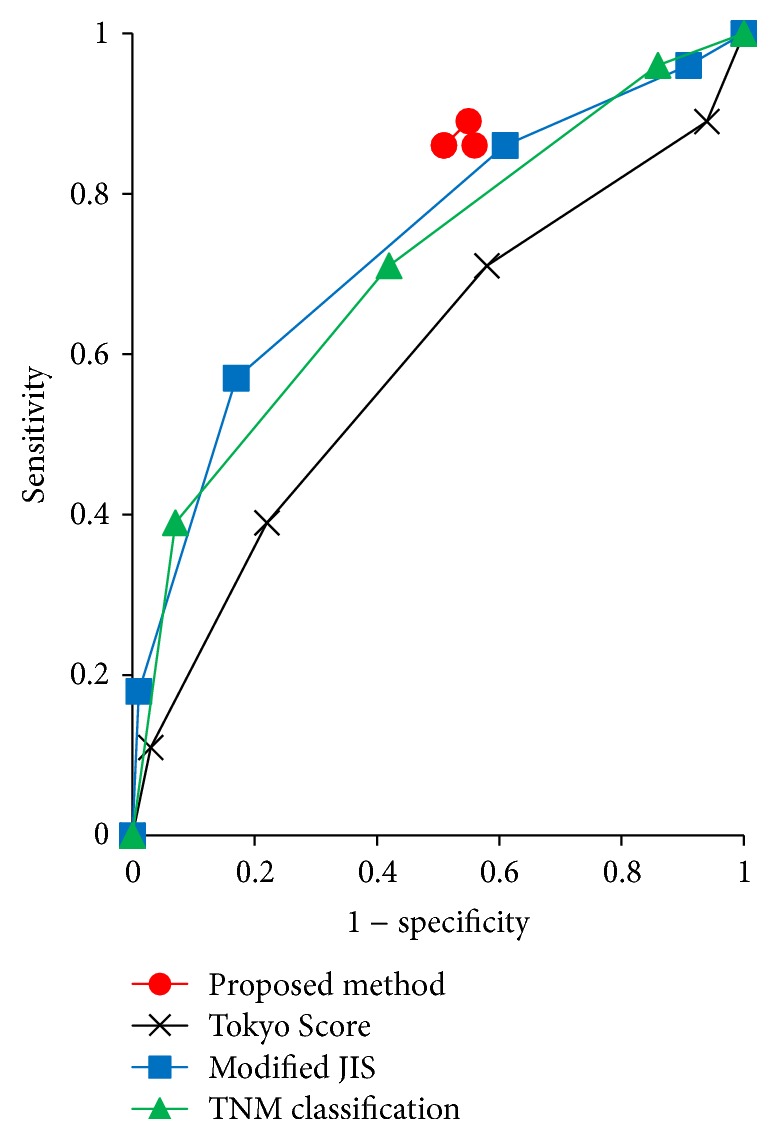
ROC curve.

Discrimination performances were compared between the discrete Bayes classifier and existing liver scoring systems. The classifier was evaluated by well-known indices such as *F*1 and the diagnostic odds ratio [23, 24], and the results are shown in Table 5. Furthermore, the results of the ROC analysis are shown in Figure 6. The discrete Bayes classifier does not use a cutoff value but instead constructs ROC curves to determine sensitivity and specificity by changing the markers one by one from 3 to 6. Additionally, between the marker numbers of 3 and 4, the sensitivity and specificity were the same at 0.86 and 0.49, respectively.

#### 3.3.4. Computational Complexity

**Figure 7:**
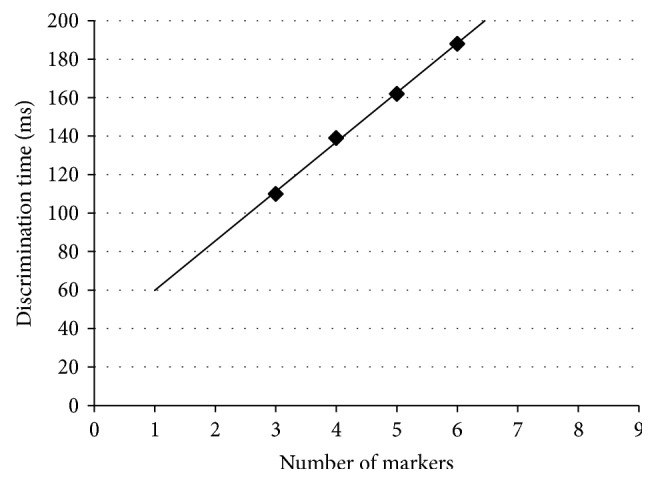
Relation between the number of markers and discrimination time.

The relationship between the number of markers and CPU time as the number of markers is changed one by one from 3 to 6 is shown in Figure 7, suggesting that linear computational complexity was observed.

### 3.4. Discussion

Based on the results of this experiment, it was revealed that a combination of 4 markers (tumor number × tumor size, vp, ICG, and liver damage) selected from among the 10 candidates was optimal. Next, when the influence of the training sample size on discrimination performance was investigated, as shown in Figure 5, the number of training samples almost converged to 100 from the viewpoint of the sensitivity. The discrimination performance was compared with that of three existing representative liver scoring systems. Discrimination performance of the discrete Bayes decision rule using the optimal combination of markers against test samples showed a sensitivity of 0.86 and a specificity of 0.49, as shown in Table 5(a). The discrete Bayes classifier achieved high sensitivity, which is an important indicator of the prediction of early recurrence, and the decrease in specificity was smaller than that of the existing scoring systems. Because we tried not to miss cancer recurrence, we adopted an evaluation standard in which sensitivity is maximum by maintaining the specificity at a certain level.

Meanwhile, in terms of accuracy and the *F*1 measure, we assumed that the numbers of recurrence test samples and nonrecurrence test samples were the same. Then, using 28 recurrence test samples and 28 nonrecurrence test samples that were derived by the resampling method, we independently reevaluated them 100 times. The results are shown in Table 5(b). From Table 5(b), we see that if the numbers of test samples in two classes are the same, this method is superior to the existing scoring methods in terms of the *F*1 measure. Next, as shown in Figure 6, an ROC curve was constructed using the data shown in Table 5(a), which shows that the curve for the proposed method is located above the curves of the existing scoring methods, suggesting that the proposed method is better than the existing scoring methods. In addition, when the number of markers was greatly changed, the specificity and sensitivity did not change significantly. Finally, we performed calculation experiments showing that as the number of markers is increased, the discrimination time also increases in linear order, which indicates that this method has an advantage in computational cost for big data such as methylation or genes that have several hundreds of thousands and several tens of thousands of candidate markers, respectively.

In addition, we point out an advantage of the discrete Bayes classifier over the existing scoring systems. Because the existing scoring systems require the use of specific markers, they cannot be used when the data of the markers are insufficient. However, the proposed technique can be used by selecting an optimal combination of markers from the laboratory data that a patient already has. Moreover, the physician is presented with the markers that should be added to improve discrimination performance for each patient. In this way, on the basis of the technique proposed here, the best personalized medicine can be expected.

## 4. Conclusion

In this paper, a discrete Bayes decision rule that predicts early recurrence of highly refractory liver cancer with a high degree of accuracy was proposed. This discrete Bayes decision rule can deal not only with qualitative data but also with quantitative data by discretization. Discrimination experiments enabled us to predict early recurrence of liver cancer with higher sensitivity than that of the Tokyo Score, the Modified JIS, and the TNM classification, which are existing representative scoring systems. Realization of personalized medicine via this discrete Bayes decision rule may be expected.

One of the main limitations of this paper was the use of a small amount of sample data from a single institution. This limited the evaluation of the performance. Further study is needed to evaluate the proposed method using data collected from other institutions. In addition, this method uses the hypothesis of independence without discussion to simplify the calculations. Although verification of independence is difficult, a study on independence such as the adoption of Bayesian networks will be a future challenge. Also, because the discrimination ability of this method depends on the cutoff value used when marker values are discretized, optimization will also be an interesting challenge.

## Figures and Tables

**Table 1:** Divisions and numbers of patients with each marker.

Division of markers	*ω*_1_	*ω*_2_
*x*_1(1)_	*n*_1(1)_^1^	*n*_1(1)_^2^
*x*_1(2)_	*n*_1(2)_^1^	*n*_1(2)_^2^

*x*_2(1)_	*n*_2(1)_^1^	*n*_2(1)_^2^
*x*_2(2)_	*n*_2(2)_^1^	*n*_2(2)_^2^
*x*_2(3)_	*n*_2(3)_^1^	*n*_2(3)_^2^

⋮	⋮	⋮

*x*_*j*(*r*_*j*_)_	*n*_*j*(*r*_*j*_)_^1^	*n*_*j*(*r*_*j*_)_^2^

⋮	⋮	⋮

*x*_*d*(*r*_*d*_)_	*n*_*d*(*r*_*d*_)_^1^	*n*_*d*(*r*_*d*_)_^2^

⋮	⋮	⋮

**Table 2:** Division and number of training samples in each class.

Division of markers	Number of samples	
	29	89
	Recurrence within 1 year	Nonrecurrence within 1 year
*x*_1(1)_ ALB > 3.5	15	60
*x*_1(2)_ ALB ≤ 3.5	14	29

*x*_2(1)_ Tumor number × tumor size < 4	6	47
*x*_2(2)_ Tumor number × tumor size 4~9	11	29
*x*_2(3)_ Tumor number × tumor size > 9	12	13

*x*_3(1)_ vp+	10	18
*x*_3(2)_ vp−	19	71

*x*_4(1)_ ICG < 15	14	44
*x*_4(2)_ ICG ≥ 15	15	45

*x*_5(1)_ vv+	9	16
*x*_5(2)_ vv−	20	73

*x*_6(1)_ Number of platelets ≥ 10	16	70
*x*_6(2)_ Number of platelets < 10	13	19

*x*_7(1)_ PT ≥ 80	18	67
*x*_7(2)_ PT < 80	11	22

*x*_8(1)_ Bilirubin < 1	17	61
*x*_8(2)_ Bilirubin ≥ 1	12	28

*x*_9(1)_ Degree of differentiation non-por	25	79
*x*_9(2)_ Degree of differentiation por	4	10

*x*_10(1)_ Liver damage A	15	60
*x*_10(2)_ Liver damage B	14	29

**Table:** (a) Breakdown of training samples

Virus type	Recurrence within 1 year	Nonrecurrence within 1 year
B	6	16
C	18	56
Samples that are neither B type nor C type	5	17

Total number of samples	29	89

**Table:** (b) Breakdown of test samples

Virus type	Recurrence within 1 year	Nonrecurrence within 1 year
B	6	16
C	17	55
Samples that are neither B type nor C type	5	17

Total number of samples	28	88

**Table 4:** Optimal combinations of markers per number of markers and their discrimination performances obtained using training samples.

Number of markers	Sensitivity	Specificity	Youden index	Combination of markers					
3	0.79	0.50	0.29	Tumor number × tumor size	vp	Liver damage			

4	0.80	0.50	0.30	Tumor number × tumor size	vp	ICG	Liver damage		

5	0.75	0.50	0.25	ALB	Tumor number × tumor size	vv	Degree of differentiation	Liver damage	

6	0.74	0.51	0.24	ALB	Tumor number × tumor size	vp	ICG	Degree of differentiation	Liver damage

7	—	—	—						
8	—	—	—						
9	—	—	—						

**Table:** (a) Results using 28 recurrence test samples and 88 nonrecurrence test samples

Index	Proposed method	Modified JIS with 3	TNM classification with 2	Tokyo score with 2
Accuracy	0.58	0.77	0.34	0.49
Sensitivity, recall	0.86	0.57	0.96	0.71
Specificity	0.49	0.83	0.14	0.42
*F*1 measure	0.49	0.54	0.41	0.40
Youden index	0.35	0.40	0.10	0.13
Diagnostic odds ratio	5.73	6.49	4.26	1.81

**Table:** (b) Results using 28 test samples/class obtained by resampling

Index	Proposed method	Modified JIS with 3	TNM classification with 2	Tokyo score with 2
Accuracy	0.67 [0.66, 0.69]	0.70 [0.69, 0.71]	0.55 [0.54, 0.56]	0.57 [0.55, 0.58]
Sensitivity, recall	0.86	0.57	0.96	0.71
Specificity	0.49 [0.46, 0.52]	0.83 [0.81, 0.85]	0.14 [0.12, 0.16]	0.42 [0.39, 0.44]
*F*1 measure	0.73 [0.72, 0.73]	0.66 [0.65, 0.67]	0.68 [0.68, 0.69]	0.62 [0.62, 0.63]
Youden index	0.35 [0.32, 0.37]	0.40 [0.38, 0.42]	0.10 [0.08, 0.12]	0.13 [0.11, 0.16]
Diagnostic odds ratio	6.03 [5.39, 6.67]	7.88 [6.22, 9.53]	4.62 [3.86, 5.38]	1.95 [1.74, 2.15]

## References

[1] Iizuka N., Hamamoto Y., Tsunedomi R., Oka M. (2008). Translational microarray systems for outcome prediction of hepatocellular carcinoma. *Cancer Science*.

[2] Tateishi R., Yoshida H., Shiina S. (2005). Proposal of a new prognostic model for hepatocellular carcinoma: an analysis of 403 patients. *Gut*.

[3] Ikai I., Takayasu K., Omata M. (2006). A modified Japan integrated stage score for prognostic assessment in patients with hepatocellular carcinoma. *Journal of Gastroenterology*.

[4] Minagawa M., Ikai I., Matsuyama Y., Yamaoka Y., Makuuchi M. (2007). Staging of hepatocellular carcinoma: assessment of the Japanese TNM and AJCC/UICC TNM systems in a cohort of 13,772 patients in Japan. *Annals of Surgery*.

[5] Henderson J. M., Sherman M., Tavill A., Abecassis M., Chejfec G., Gramlich T. (2003). AHPBA/AJCC consensus conference on staging of hepatocellular carcinoma: consensus statement. *HPB*.

[6] Golub T. R., Slonim D. K., Tamayo P. (1999). Molecular classification of cancer: class discovery and class prediction by gene expression monitoring. *Science*.

[7] Khan J., Wei J. S., Ringnér M. (2001). Classification and diagnostic prediction of cancers using gene expression profiling and artificial neural networks. *Nature Medicine*.

[8] Furey T. S., Cristianini N., Duffy N., Bednarski D. W., Schummer M., Haussler D. (2000). Support vector machine classification and validation of cancer tissue samples using microarray expression data. *Bioinformatics*.

[9] Iizuka N., Oka M., Hamamoto Y. (2003). Oligonucleotide microarray for prediction of early intrahepatic recurrence of hepatocellular carcinoma after curative resection. *The Lancet*.

[10] Tibshirani R., Hastie T., Narasimhan B., Chu G. (2002). Diagnosis of multiple cancer types by shrunken centroids of gene expression. *Proceedings of the National Academy of Sciences of the United States of America*.

[11] Lee K. E., Sha N., Dougherty E. R., Vannucci M., Mallick B. K. (2003). Gene selection: a Bayesian variable selection approach. *Bioinformatics*.

[12] Xiong M., Li W., Zhao J., Jin L., Boerwinkle E. (2001). Feature (gene) selection in gene expression-based tumor classification. *Molecular Genetics and Metabolism*.

[13] Guyon I., Weston J., Barnhill S., Vapnik V. (2002). Gene selection for cancer classification using support vector machines. *Machine Learning*.

[14] Kourou K., Exarchos T. P., Exarchos K. P., Karamouzis M. V., Fotiadis D. I. (2015). Machine learning applications in cancer prognosis and prediction. *Computational and Structural Biotechnology Journal*.

[15] Iizuka N., Oka M., Sakaida I. (2011). Efficient detection of hepatocellular carcinoma by a hybrid blood test of epigenetic and classical protein markers. *Clinica Chimica Acta*.

[16] Parthiban L., Subramanian R. (2009). CANFIS—a computer aided diagnostic tool for cancer detection. *Journal of Biomedical Science and Engineering*.

[17] Jain A. K., Duin R. P. W., Mao J. (2000). Statistical pattern recognition: a review. *IEEE Transactions on Pattern Analysis and Machine Intelligence*.

[18] Duda R. O., Hart P. E., Stork D. G. (2001). *Pattern Classification*.

[19] Jain A. K., Dubes R. C., Chen C. (1987). Bootstrap techniques for error estimation. *IEEE Transactions on Pattern Analysis and Machine Intelligence*.

[20] Tokumitsu Y., Tamesa T., Matsukuma S. (2015). An accurate prognostic staging system for hepatocellular carcinoma patients after curative hepatectomy. *International Journal of Oncology*.

[21] Webb A. R. (2002). *Statistical Pattern Recognition*.

[22] Youden W. J. (1950). Index for rating diagnostic tests. *Cancer*.

[23] van Rijsbergen C. J. (1974). Foundation of evaluation. *Journal of Documentation*.

[24] Powers D. M. W. (2011). Evaluation: from precision, recall and F-measure to ROC, informedness, markedness & correlation. *Journal of Machine Learning Technologies*.

